# Supplementary material for: Elucidation of Spartina dimethylsulfoniopropionate synthesis genes enables engineering of stress tolerant plants
Source: Nat Commun. 2024 Oct 9;15:8568. doi: 10.1038/s41467-024-51758-z (PMC11464771; doi:10.1038/s41467-024-51758-z)
Supplement: Supplementary file 1 — Supplementary Information [file 41467_2024_51758_MOESM1_ESM.pdf]

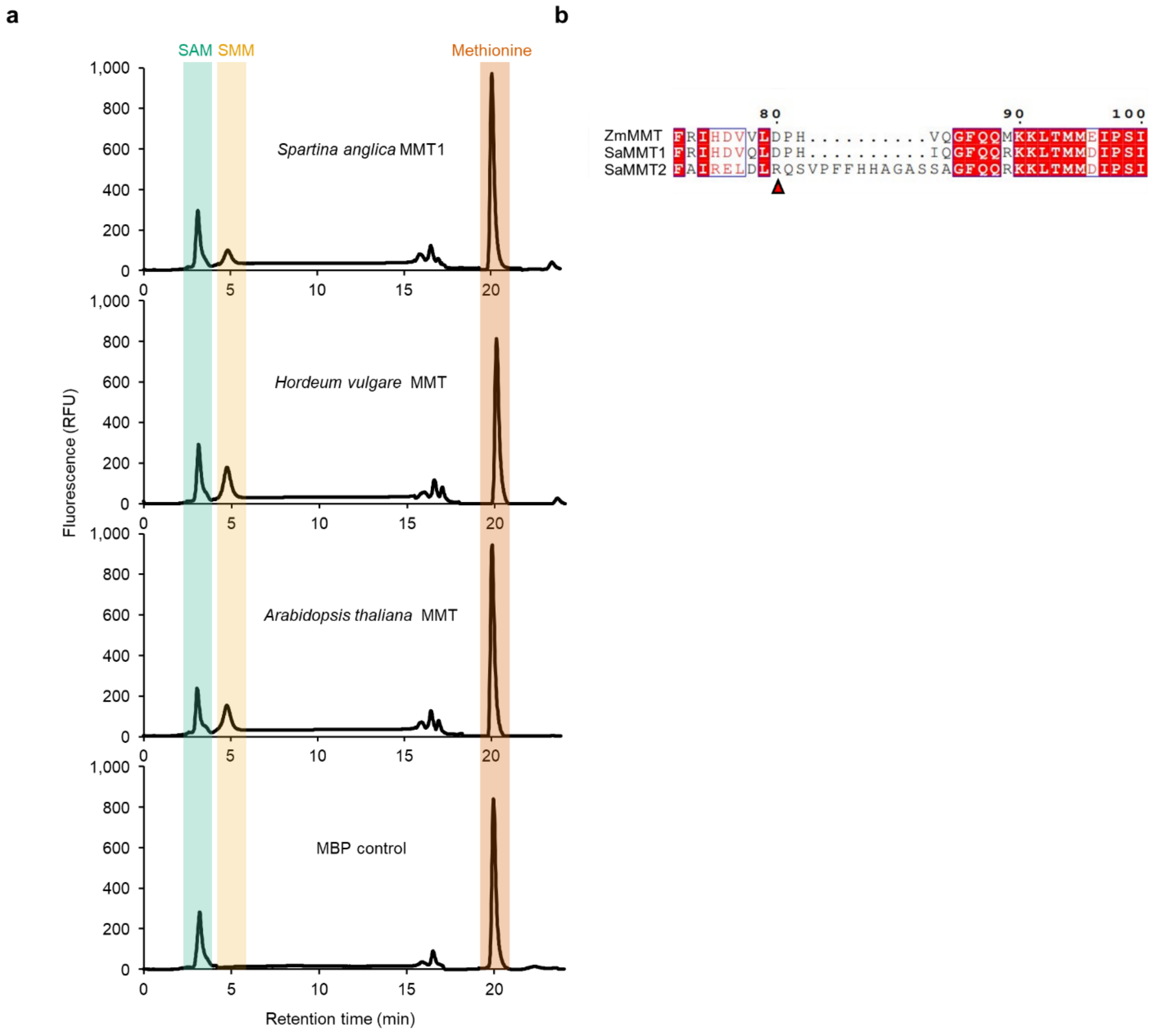

Supplementary Figure 1: *SaMMT1* is a functional methionine *S*-methyltransferase.
a, HPLC traces of recombinant SaMMT1, HvMMT, AtMMT incubated with methionine and SAM, demonstrating the production of SMM from methionine and SAM of all three enzymes. MBP control is purified maltose binding protein incubated with MMT assay reaction mix. **b,** Alignment of MMT from *Zea mays* against SaMMT sequences demonstrating insertion in SaMMT2 SAM binding domain. Red triangle indicates the beginning of the domain.

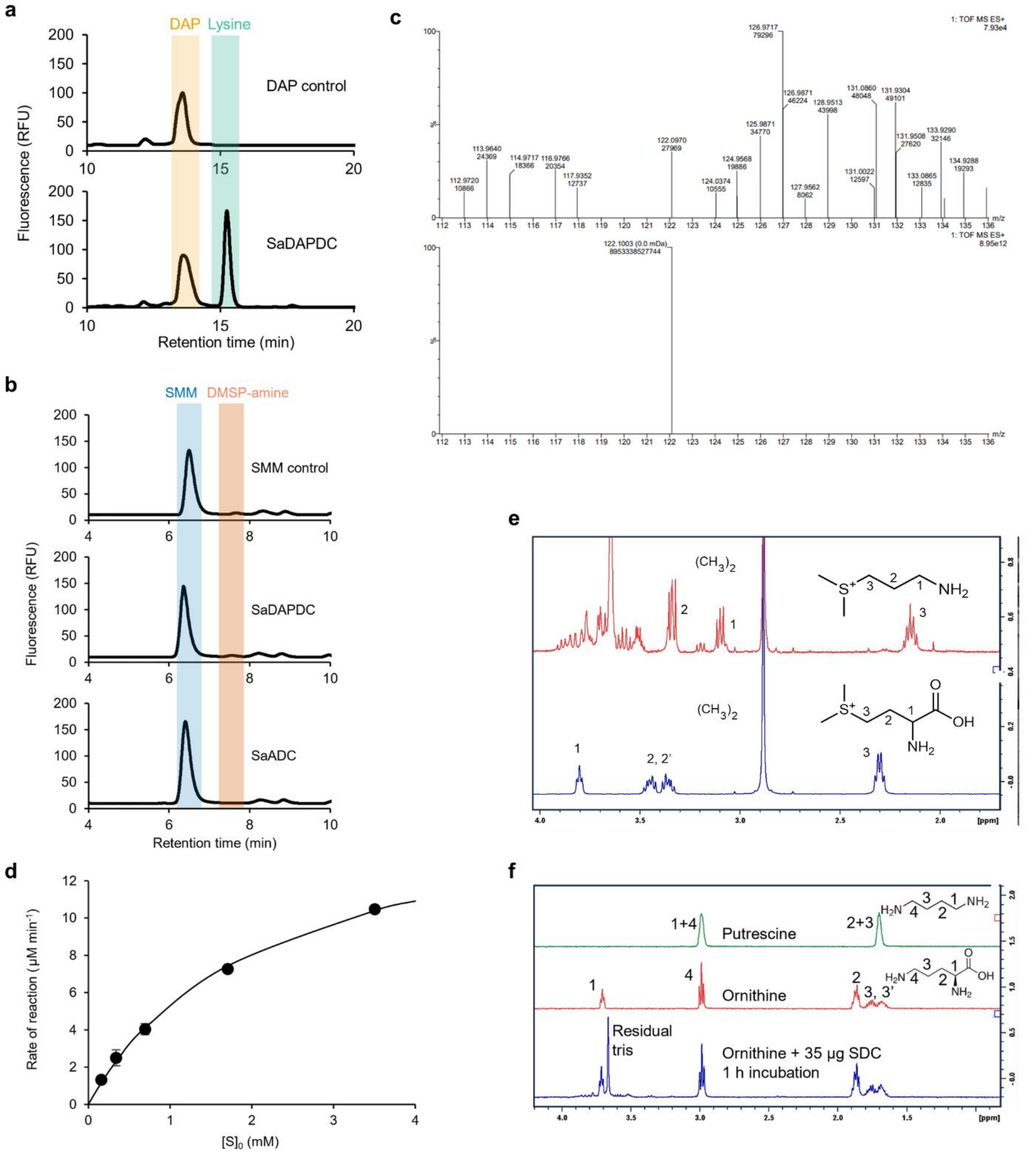

Supplementary Figure 2: SaODC has SDC activity, whereas SaDAPDC and SaADC do not.

a, HPLC traces of diaminopimelate retention time, and recombinant SaDAPDC incubated with diaminopimelate (DAP), demonstrating this enzyme is functional *in vitro*. **b,** HPLC traces shows SMM retention time, and incubation of SaDAPDC and SaADC with SMM, which show no activity with this substrate despite being *in vitro* functional enzymes. **c,** LC-MS total ion chromatogram of products of SaODC incubation with SMM, demonstrating presence of DMSP-amine (top) and expected mass of protonated form of DMSP-amine (bottom). **d,** Reaction rate of SaSDC with varying concentrations of SMM, used to calculate K_m and V_{max} of the enzyme. **e,** NMR assignment of SMM (blue) and DMSP-amine (red). For quantification of the reaction rate in panel d, peak number three (corresponding to the CH_2 adjacent to the sulphur) was used. **f,** NMR assignment of ornithine (red) and putrescine (green). No reduction is observed in peak 1 and 2 in the blue spectra, after 1 hour of incubation with 35 μg of SDC, suggesting limited activity on this substrate.

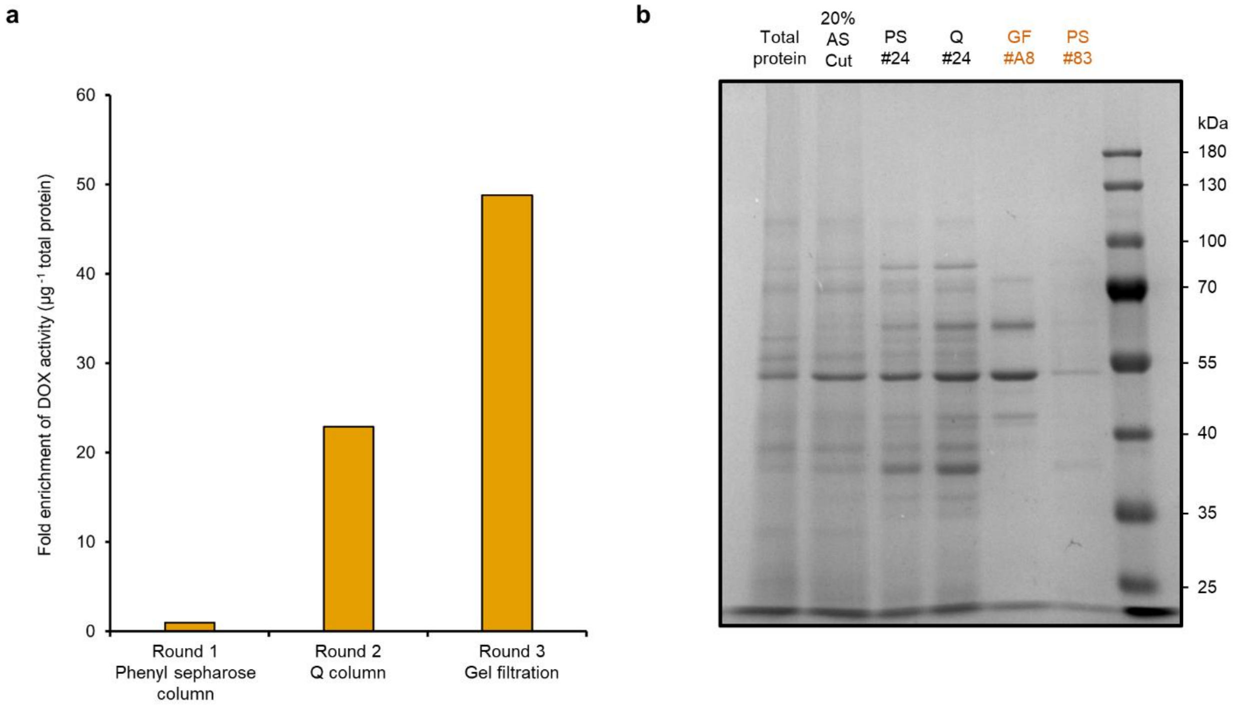

Supplementary Figure 3: SaDOX and SaCAO1 have major and minor DOX activities, respectively.
a, Fold enrichment of DOX activity in total protein extracts from *S. anglica* leaves following successive rounds of chromatography. **b,** SDS-PAGE gel of 3 μg *S. anglica* leaf protein fractions following each step of the activity tracking process, demonstrating removal of proteins with each round. Highlighted lanes are the samples which were sent for mass spectrometry analysis. AS: ammonium sulfate, PS: phenyl sepharose, Q: Q strong anion exchange, GF: gel filtration. Number following `#` refers to the fraction number after Akta separation. Source data are provided as a Source Data file.

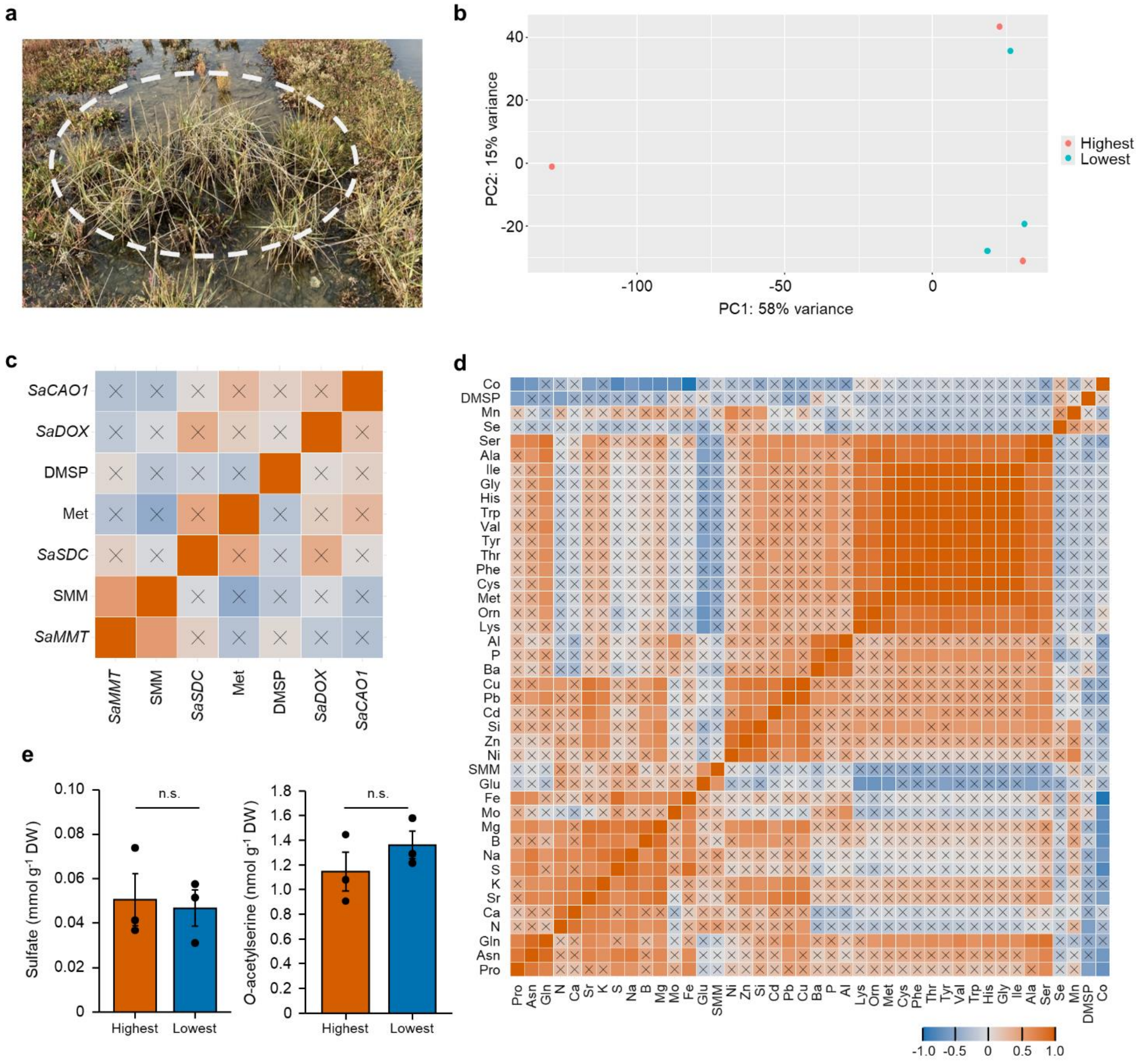

Supplementary Figure 4: DMSP accumulation in *Spartina anglica*.
a, Example clump of *S. anglica* growing at Siffkey, Norfolk, UK. **b,** PCA of RNA-seq libraries generated from highest and lowest DMSP accumulating *S. anglica*. Libraries cluster, but not based on DMSP accumulation. **c,** Heatmap of pairwise Pearson correlations between expression level of *SaMMT*, *SaSDC* and *SaDOX* and DMSP, methionine (Met) and SMM, showing that gene expression levels do not correlate with DMSP accumulation, but *SaMMT* expression does correlate with SMM accumulation. **d,** Heatmap of pairwise Pearson correlations as in Figure 2e but elements represent measurements from soil rather than leaf tissue. Amino acid measurements derive from leaf, as do DMSP measurements. This shows the relationship between nutrient and heavy metal levels in the soil and DMSP and amino acid accumulation in leaves. Crosses indicate that a relationship is not significant. **e,** Sulfate and *O*-acetylserine measurements in leaf tissues from highest and lowest DMSP-accumulating clumps of *S. anglica*. n.s. denotes no statistically significant difference in a two-tailed student's t-test. Source data are provided as a Source Data file.

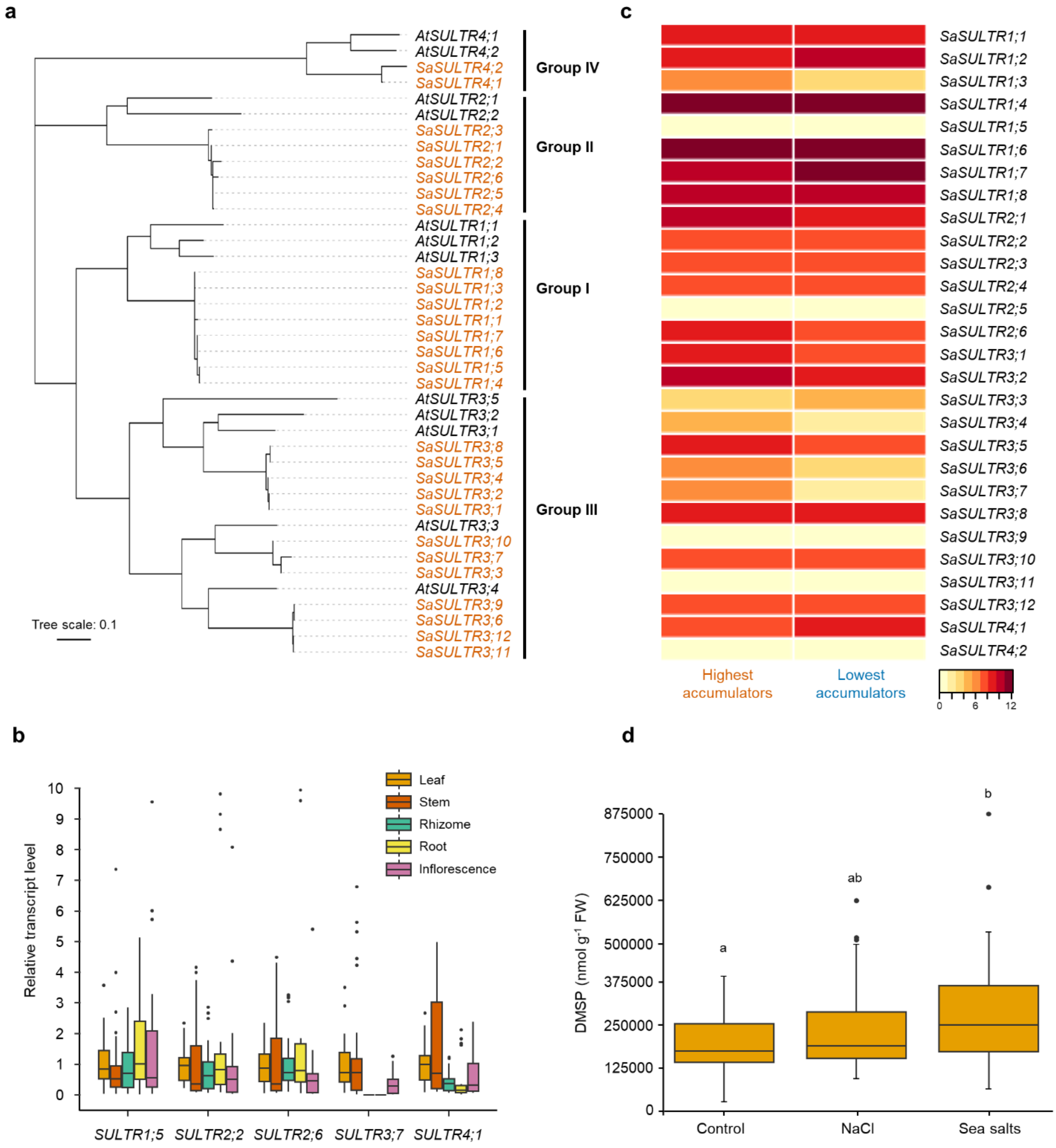

Supplementary Figure 5: Sulfate transporters are expressed in *Spartina anglica*.
a, Phylogenetic tree of SULTRs from *Arabidopsis thaliana* (black) and *S. anglica* (orange). **b,** RT-qPCR of selected *S. anglica SULTR* genes in denoted tissues, with values for leaf tissue normalised to 1 for each gene. *SULTR3;7* expression was not detected (n.d.) in rhizome or root tissue. Data represent mean ± one standard error from n = 4 independent biological replicates. **c,** Heatmap of read counts for *SULTR* genes in leaf tissues from highest and lowest DMSP-accumulating clumps of *S. anglica*, with values representing Log2(read count +1) where 1 is a psuedocount. **d,** DMSP accumulation in glasshouse-grown *S. anglica* plants watered with deionised water (control), 500 mM NaCl, or 35 PSU seas salts. Data represent mean from six independent biological replicates, with three technical replicate measurements made per sample for each time-point (n = 18). Boxplots denote median (centre line), upper and lower quartiles (box limits), the interquartile range (whiskers) and outliers (black dots). Letters indicate statistical significance after one-way ANOVA followed by Tukey test. Source data are provided as a Source Data file.

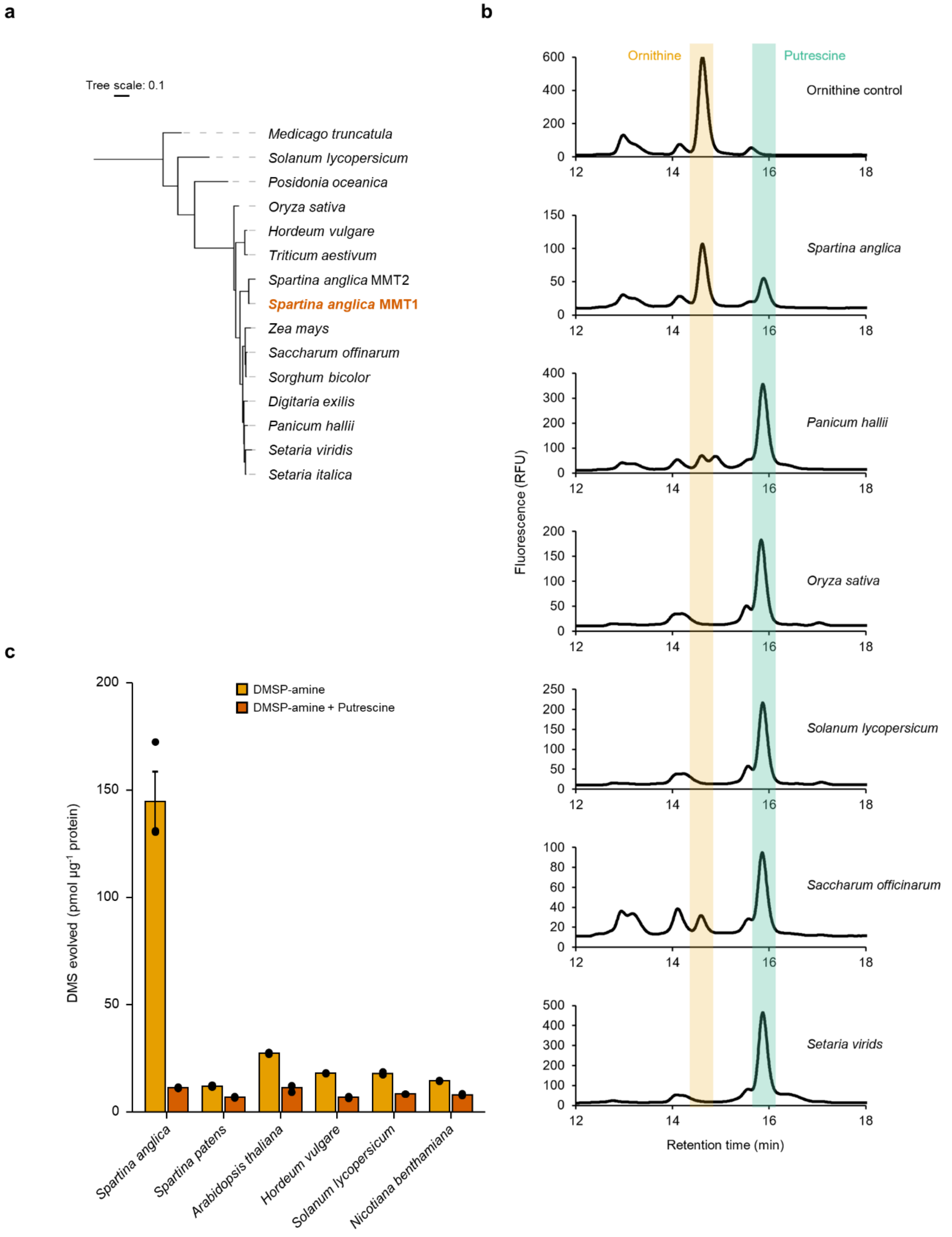

Supplementary Figure 6: Other plant species have MMT enzymes, DOX activity and functional ODC enzymes.
a, Phylogenetic tree of MMT proteins from higher plants rooted against bacterial mmtN, showing that SaMMT1 and SaMMT2 closely resemble other plant MMT enzymes. **b,** HPLC traces of recombinant SaSDC and homologous ODC enzymes incubated with ornithine, demonstrating all enzymes have *in vitro* functionality, and that *SaSDC* works less well with this substrate. **c,** DOX assays performed on protein extracts from the denoted species, demonstrating *S. anglica* has enhanced DOX activity per unit protein, and in all cases DOX enzymes are inhibited by addition of excess putrescine. Data represent mean ± one standard error from n ≥ 3 independent biological replicates. Source data are provided as a Source Data file.

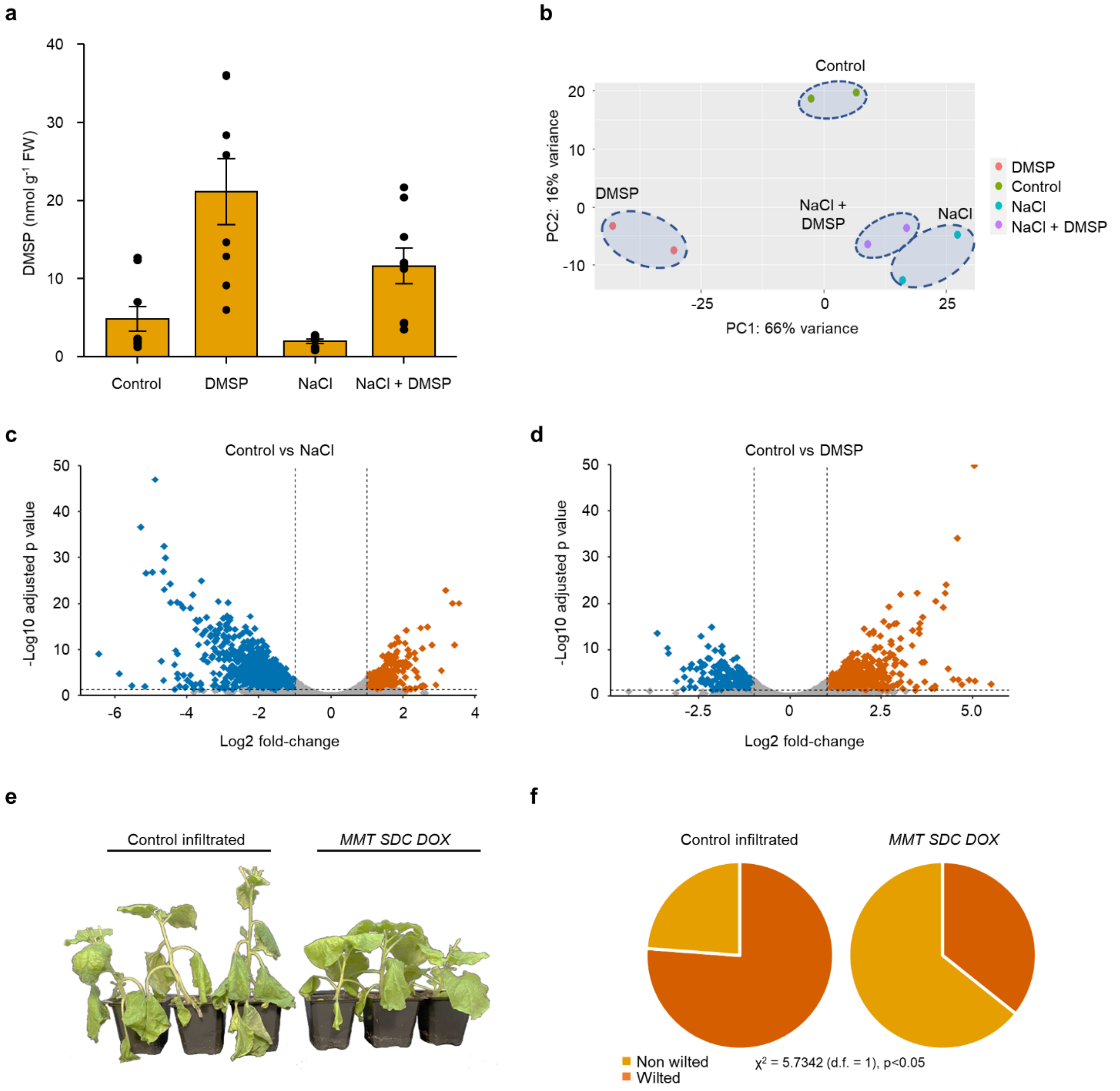

Supplementary Figure 7: Root uptake of DMSP or over-expression of *S. anglica MMT*, *SDC* and *DOX* have protective effects against salt stress and drought.

a, GC measurements of leaf tissue of tomato shown in Figure 4a, demonstrating that DMSP is taken up by roots and accumulates in leaf tissue. **b,** PCA plot of RNA-seq performed on tomato experiment presented in Figure 4, showing treatment groups cluster together. **c,** Volcano plot demonstrating broad transcriptomic changes in tomato following NaCl treatment. Each dot represents a gene, with blue dots indicating genes that are downregulated in NaCl treatment, red dots representing upregulation, and grey dots denoting genes that are not significantly differentially expressed. Dotted lines indicate significance thresholds. **d,** Volcano plot as in panel **c**, but comparing DMSP treated vs untreated tomato plants. **e,** Image of *N. benthamiana* plants under drought conditions, in which every leaf has either been infiltrated with *Agrobacterium tumefaciens* containing no plasmid (control infiltrated) or containing a plasmid to over-express *S. anglica MMT, SDC* and *DOX.* **f,** Pie charts representing the frequency of wilted vs non wilted leaves from plants treated as described in panel e. Data represent mean ± one standard error from n ≥ 3 independent biological replicates. Source data are provided as a Source Data file.

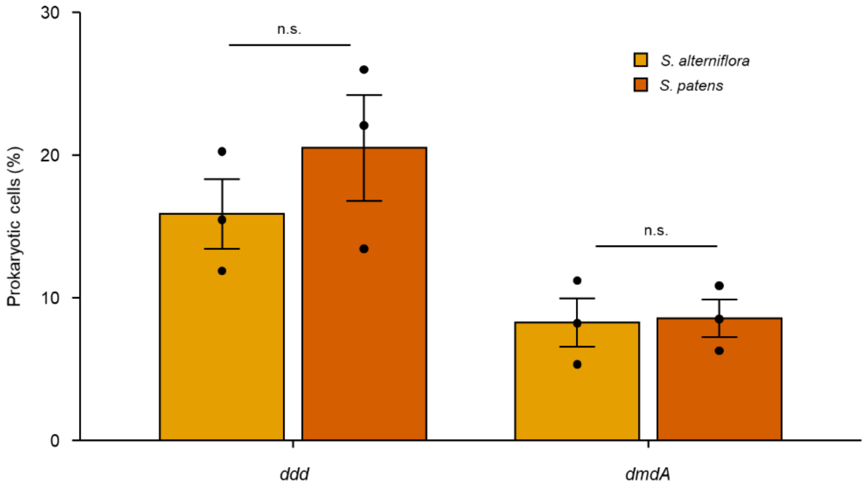

Supplementary Figure 8: Abundance of *ddd* and *dmdA* genes in the sediments of *Spartina alterniflora* (high DMSP producer) and *S. patens* (low DMSP producer).
Relative abundance of the nine prokaryotic DMSP lyase *ddd* genes and the DMSP demethylation *dmdA* gene. Data represent mean ± one standard error from three independent biological replicates (n = 3). n.s. denotes no statistically significant difference after one-way ANOVA followed by Tukey test. Source data are provided as a Source Data file.
