# Supplementary material for: Elucidation of Spartina dimethylsulfoniopropionate synthesis genes enables engineering of stress tolerant plants
Source: Nat Commun. 2024 Oct 9;15:8568. doi: 10.1038/s41467-024-51758-z (PMC11464771; doi:10.1038/s41467-024-51758-z)
Supplement: Supplementary file 3 — Description of Additional Supplementary Files [file 41467_2024_51758_MOESM3_ESM.pdf]

Description of Additional Supplementary Files

Supplementary Data 1: BADH / DADH candidates identified from *S. anglica* transcriptome.

Table of candidate BADH / DADH genes identified from BLAST analysis of *S. anglica* transcriptome. cDNA sequence contains the complete transcript sequence from transcriptome assembly, including 5' and 3' UTRs.

Supplementary Data 2: MMT, SDC and DOX candidates identified in *S. anglica* transcriptome.

Candidate genes identified through BLAST analysis of *S. anglica* transcriptome using entry in Interpo ID column as subject sequence. Highlighted rows indicate candidate genes which were identified to have the correct activity.

Supplementary Data 3: Mass spectrometry data identifying SaDOX.

Protein LC-MS/MS results from sample 'GF#A8' (Supplementary Figure 3b), which contained SaDOX (highlighted in red). Protein identification percentage represents the confidence of the BLAST assignment and exclusive unique peptide count represents the number of peptides which mapped only to the assigned protein. Exclusive unique spectrum count represents the number of spectra unique to the assigned protein and the exclusive spectra count is a total of all spectra assigned specifically to the protein. Percentage of total spectra indicates abundance of protein relative to all others in the sample, percentage sequence coverage represents the total length of the protein identified in the LC-MS run, and the length is simply the total length of the protein.

Supplementary Data 4: Genes differentially expressed between highest and lowest DMSP-accumulating clumps of *S. anglica.*

Differential expression analysis of *S. anglica* clumps with highest vs. lowest accumulation of DMSP calculated by DESeq2. baseMean is the average of the normalised counts taken across all samples, log2FoldChange is the log-2 fold change, a positive value indicates an increase in high accumulators, and a negative value a decrease in high accumulators (relative to low). lfcSE is the standard error of the log2 fold change estimate, stat is Wald statistic, p-value is the result of Wald test and p-adj is p-value adjusted for multiple testing using the Benjamini-Hochberg test.

Supplementary Data 5: Homologs of *MMT, SDC, DOX, CAO1, EF1A* and *ACT8* from *S. anglica, A. thaliana, S. lycopersicum, N benthamiana* and *H. vulgare.*

Table of homologs of *MMT, SDC, DOX, CAO1, EF1A* and *ACT8* used to produce Figure 3f. Contig refers to the contig name in the transcriptome, sequence is the cDNA sequence taken from reference transcriptome and the study indicates from where the data was obtained. In the case of *S. patens*, sequence is CDS sequence, obtained by cloning and sanger sequencing from gDNA.

Supplementary Data 6: GO enrichment of DMSP responsive genes under NaCl stress in *S. lycopersicum.*

Gene ontology enrichment analysis of genes differentially expressed between NaCl and NaCl + DMSP treated tomato computed using ShinyGO with species set to *Solanum lycopersicum* STRINGdb. Enrichment FDR is the p-value for the Fold Enrichment, corrected using the False Discovery Rate (FDR) method. Pathway Genes refers to the number of genes annotated in the database with that particular term; this number is used along with the number of DE genes with the corresponding GO-term (reported in Genes column) to calculate Fold Enrichment.

Supplementary Data 7: Raw read information and copy number of ten single copy marker genes, nine DMSP lyase *ddd* genes and DMSP demethylation *dmdA* gene. COG ID number is taken from the Clusters of Orthologous Genes database.

Supplementary Data 8: Oligonucleotide sequences used in this study.
