# Supplementary material for: Elucidation of Spartina dimethylsulfoniopropionate synthesis genes enables engineering of stress tolerant plants
Source: Nat Commun. 2024 Oct 9;15:8568. doi: 10.1038/s41467-024-51758-z (PMC11464771; doi:10.1038/s41467-024-51758-z)
Supplement: Supplementary file 12 — Reporting Summary [file 41467_2024_51758_MOESM12_ESM.pdf]

nature portfolio

Corresponding author(s): Jonathan Todd & Ben Miller

Last updated by author(s): Aug 2, 2024

Reporting Summary

Nature Portfolio wishes to improve the reproducibility of the work that we publish. This form provides structure for consistency and transparency in reporting. For further information on Nature Portfolio policies, see our Editorial Policies and the Editorial Policy Checklist.

Statistics

For all statistical analyses, confirm that the following items are present in the figure legend, table legend, main text, or Methods section.

n/a	Confirmed	
☐	☒	The exact sample size (*n*) for each experimental group/condition, given as a discrete number and unit of measurement
☐	☒	A statement on whether measurements were taken from distinct samples or whether the same sample was measured repeatedly
☐	☒	The statistical test(s) used AND whether they are one- or two-sided<br>*Only common tests should be described solely by name; describe more complex techniques in the Methods section.*
☐	☒	A description of all covariates tested
☐	☒	A description of any assumptions or corrections, such as tests of normality and adjustment for multiple comparisons
☐	☒	A full description of the statistical parameters including central tendency (e.g. means) or other basic estimates (e.g. regression coefficient) AND variation (e.g. standard deviation) or associated estimates of uncertainty (e.g. confidence intervals)
☐	☒	For null hypothesis testing, the test statistic (e.g. *F*, *t*, *r*) with confidence intervals, effect sizes, degrees of freedom and *P* value noted<br>*Give P values as exact values whenever suitable.*
☒	☐	For Bayesian analysis, information on the choice of priors and Markov chain Monte Carlo settings
☒	☐	For hierarchical and complex designs, identification of the appropriate level for tests and full reporting of outcomes
☐	☒	Estimates of effect sizes (e.g. Cohen's *d*, Pearson's *r*), indicating how they were calculated

Our web collection on statistics for biologists contains articles on many of the points above.

Software and code

Policy information about availability of computer code

Data collection	For HPLC, peaks were integrated in Dionex Chromeleon v.6 software and Jasco ChromNav v.1 software (as detailed in the Methods section).
Data analysis	For differential expression analyses, adapters were trimmed from sequencing reads using Trim Galore. For Spartina anglica, a reference transcriptome was built using Trinity under default settings. Reads were aligned using HISAT2, quantified using Slamon, and statistical testing of quantified reads was performed using DESeq2. All gene ontology enrichments were performed using ShinyGO v0.77. For phylogenetic tree assembly, sequences were aligned using MUSCLE and the subsequent Phylip file was run through PhylML. For metagenomic datasets, data quality was assessed using FastQC v0.12.1, and assembly (MEGAHIT v1.2.9), annotation (Prodigal v2.6.3), and non-redundant protein sequence generation (CD-HIT v4.8.1) were performed. Gene relative abundances were estimated by the “metabat” method in CoverM 0.6.1. References for data analysis tools have been included. R Studio was used for all other statistical tests.

For manuscripts utilizing custom algorithms or software that are central to the research but not yet described in published literature, software must be made available to editors and reviewers. We strongly encourage code deposition in a community repository (e.g. GitHub). See the Nature Portfolio guidelines for submitting code & software for further information.

Data

Policy information about availability of data

All manuscripts must include a data availability statement. This statement should provide the following information, where applicable:

- Accession codes, unique identifiers, or web links for publicly available datasets
- A description of any restrictions on data availability
- For clinical datasets or third party data, please ensure that the statement adheres to our policy

Source data are provided as a Source Data file. The RNA-seq data generated in this study have been deposited in the NCBI SRA database under accession codes SAMN42957141 and SAMN42997936. All unique materials used are readily available from the corresponding authors upon reasonable request.

Research involving human participants, their data, or biological material

Policy information about studies with human participants or human data. See also policy information about sex, gender (identity/presentation), and sexual orientation and race, ethnicity and racism.

Reporting on sex and gender	n/a
Reporting on race, ethnicity, or other socially relevant groupings	n/a
Population characteristics	n/a
Recruitment	n/a
Ethics oversight	n/a

Note that full information on the approval of the study protocol must also be provided in the manuscript.

Field-specific reporting

Please select the one below that is the best fit for your research. If you are not sure, read the appropriate sections before making your selection.

☒ Life sciences ☐ Behavioural & social sciences ☐ Ecological, evolutionary & environmental sciences

For a reference copy of the document with all sections, see nature.com/documents/nr-reporting-summary-flat.pdf

Life sciences study design

All studies must disclose on these points even when the disclosure is negative.

Sample size	No statistical methods were used to predetermine sample size. Sample size was determined by convention in plant biology research, with a minimum of three independent biological replicates as is standard practice.
Data exclusions	No data were excluded from the analysis.
Replication	A minimum of three independent biological replicates were performed. All attempts at replication were successful.
Randomization	Allocation was now performed, therefore randomization is not applicable.
Blinding	Blinding was not performed as groups were not subjected to categorization.

Reporting for specific materials, systems and methods

We require information from authors about some types of materials, experimental systems and methods used in many studies. Here, indicate whether each material, system or method listed is relevant to your study. If you are not sure if a list item applies to your research, read the appropriate section before selecting a response.

Materials & experimental systems

n/a	Involved in the study
☒	☐ Antibodies
☒	☐ Eukaryotic cell lines
☒	☐ Palaeontology and archaeology
☐	☒ Animals and other organisms
☒	☐ Clinical data
☒	☐ Dual use research of concern
☐	☒ Plants

Methods

n/a	Involved in the study
☒	☐ ChIP-seq
☒	☐ Flow cytometry
☒	☐ MRI-based neuroimaging

Animals and other research organisms

Policy information about studies involving animals; ARRIVE guidelines recommended for reporting animal research, and Sex and Gender in Research

Laboratory animals	The study did not involve laboratory animals.
Wild animals	The study did not involve wild animals.
Reporting on sex	n/a
Field-collected samples	n/a
Ethics oversight	n/a

Note that full information on the approval of the study protocol must also be provided in the manuscript.

Plants

Seed stocks	Spartina anglica samples were collected from the field as described in the Methods section. Wildtype Arabidopsis thaliana seeds were obtained from the Nottingham Arabidopsis Stock Centre (NASC) and tomato (Solanum lycopersicum cv. 'Micro Tom') seeds were obtained from Moles Seeds (www.wholesale.molesseeds.co.uk).
Novel plant genotypes	Transgenic plant material was generated using Agrobacterium tumefaciens via floral dip or leaf infiltration as described in the Methods section. For stable transgenics, five independent T2 lines were analysed, of which three lines were further analysed at the T3 generation.
Authentication	Transgenic plant material was generated using Agrobacterium tumefaciens via floral dip or leaf infiltration as described in the Methods section. For stable transgenics, five independent T2 lines were analysed, of which three lines were further analysed at the T3 generation.
